# Acute‑on‑chronic kidney disease (AoCKD): a critical appraisal and call for action to define boundaries and optimize management

**DOI:** 10.1080/0886022X.2026.2746268

**Published:** 2026-10-29

**Authors:** Amin R. Soliman, Karim M. Soliman, Salwa Ibrahim Mohamed, Mohamed Mostafa Elkhatib, Mohamed Momtaz, Mahmoud M. Elnokeety, Ahmed Aly, Hoda Maamoun, Tarek S. Abdelaziz, Hany Hammad, Mahmoud M. Shaheen, Rasha Ahmed Darwish, Ahmed Fayed, Dina A. Abdellatif, Nouran Hatem Samir, Ahmed S. Hegazy, Sahier El-Khashab, Kasr Alainy Nephrology group (KANG)

**Affiliations:** a Nephrology Unit, Internal Medicine Department, Kasr Alainy School of Medicine, Cairo University, Cairo, Egypt; b Division of Transplant Nephrology, Department of Medicine, Medical University of South Carolina, Charleston, SC, USA; c Medical Services, Ralph H. Johnson VA Medical Centre, Charleston, SC, USA

**Keywords:** Acute-on-chronic kidney disease, acute kidney injury, acute kidney disease, biomarkers of kidney injury, prognosis of acute kidney injury, clinical practice guidelines

## Abstract

Acute‑on‑chronic kidney disease (AoCKD) refers to an acute decline in renal function occurring in individuals with established CKD. Despite its major clinical impact—including increased mortality, accelerated CKD progression, and higher cardiovascular risk—AoCKD remains underrepresented within major guideline frameworks (KDIGO, ADQI, NICE). Our review aims to critically appraise existing guideline frameworks, definitions, biomarkers, imaging recommendations, and research gaps related to AoCKD, and to highlight priorities for future harmonization and clinical integration. We conducted a targeted literature search of PubMed, Embase, and the Cochrane Library, to identify guidelines, consensus reports, observational studies, and systematic reviews describing acute deterioration in CKD. Extracted data included definitional criteria, epidemiology, biomarker performance, imaging guidance, and prognostic outcomes. Current KDIGO, ADQI, and NICE frameworks were evaluated in parallel. We report marked heterogeneity in definitions describing acute deterioration in CKD, with terms including AoCKD, AKI‑on‑CKD, acute pre‑existing kidney dysfunction, and cROCK‑based definitions. Reported incidence varies widely (1–33%) due to inconsistent criteria. Creatinine‑based definitions show reduced prognostic accuracy in CKD populations, whereas cystatin‑C–based eGFR demonstrates superior discrimination for mortality and progression. We propose an operational definition of AoCKD that includes functional criteria and biomarker-based criteria. The definition reflects the increased mortality in this phenotype even before reaching the KDIGO-AKI threshold. In conclusion, AoCKD is a clinically significant but poorly defined state within the kidney disease continuum. Harmonized definitions, incorporation of cystatin‑C–based markers, structured imaging pathways, and integration of early biomarkers are urgently needed.

## Introduction

Acute kidney injury (AKI) and chronic kidney disease (CKD) are both highly prevalent conditions that together contribute substantially to global morbidity and mortality [1,2]. A growing body of evidence demonstrates a bidirectional relationship between the two disorders: AKI accelerates CKD progression, while CKD increases susceptibility to AKI and worsens outcomes [3].

The definition of AKI has evolved considerably over the past century, with over thirty-five historical descriptions of abrupt kidney function decline, beginning with early reports such as ‘war nephritis’ during World War [4]. The modern conceptualization of AKI was formalized in the KDIGO 2012 guideline, which established standardized diagnostic criteria based on changes in serum creatinine and urine output [5]. The term acute kidney disease (AKD) was introduced in the KDIGO Clinical Practice Guideline for Acute Kidney Injury and subsequently formalized by the ADQI 16 Workgroup. The draft KDIGO 2026 AKI/AKD Guideline defines AKD as abnormalities of kidney function and/or structure present for ≤3 months, including AKI, reduced GFR, substantial declines in kidney function, increases in serum creatinine, or markers of kidney damage.

This broadened definition reinforces AKD as a temporal framework encompassing diverse acute and subacute kidney disorders, irrespective of whether a recognized AKI episode has occurred.

Recent meta‑analyses demonstrate substantial heterogeneity in AKD definitions, contributing to wide variation in reported incidence and outcomes [6,7].

Nevertheless, AKD does not specifically distinguish acute deterioration occurring in patients with established CKD, which remains the focus of the proposed AoCKD construct.

The publication of a draft of the KDIGO 2026 Clinical Practice Guideline for Acute Kidney Injury (AKI) and Acute Kidney Disease (AKD) represents an important advance toward harmonizing definitions across the spectrum of kidney disorders [8]. The guideline is still in draft subject to receiving feedback from experts. The updated guideline provides greater clarity regarding AKI, AKD, CKD, and the role of kidney injury biomarkers. Nevertheless, patients with established CKD who experience acute deterioration remain a particularly vulnerable population with disproportionately poor renal and cardiovascular outcomes. While such patients may fulfill existing AKI or AKD criteria, specific guidance regarding phenotype identification, risk stratification, biomarker integration, imaging pathways, and management strategies in the setting of preexisting CKD remains limited. Accordingly, we believe there remains value in critically examining the concept of acute-on-chronic kidney disease (AoCKD) as a clinically important phenotype within the AKI-AKD-CKD continuum rather than as a separate disease entity. Acute deterioration occurring in patients with established CKD remains inconsistently described across the literature, using terms such as AKI-on-CKD, acute-on-preexisting kidney disease (APKD), acute-on-chronic kidney injury (ACKI), and acute-on-chronic kidney disease (AoCKD). This heterogeneity has adversely affected epidemiological comparisons and consensus development.

Table 1 outlines and criteria for defining AKI, AKD and CKD.

**Table 1.** Definitions across the guidelines.

Parameter	AKI (KDIGO 2026)	AKD (KDIGO 2026)	CKD (KDIGO 2024)
Timeframe	≤7 days	>7 to <90 days	≥90 days
Definition	Increase in serum creatinine, decrease in urine output and/or evidence of kidney injury according to KDIGO criteria	AKI, or abnormalities of kidney function and/or structure with duration <3 months	Abnormalities of kidney structure or function present for ≥3 months, with implications for health
Initiating Event Required	Yes	Not necessarily	Not applicable
Baseline CKD Required	No	No	Yes
Structural Biomarkers Considered	Yes (where validated)	Yes	Yes
Primary Clinical Challenge	Recognition of acute injury	Recognition and monitoring of persistent acute dysfunction	Staging and long-term risk assessment

AoCKD represents a high-risk clinical phenotype characterized by acute deterioration in kidney function occurring in patients with preexisting CKD. Although it exists within the AKI-AKD-CKD continuum recognized by KDIGO, it remains inconsistently defined and inadequately addressed in current guideline frameworks (Figure 1) [5,9,10].

**Figure 1.**
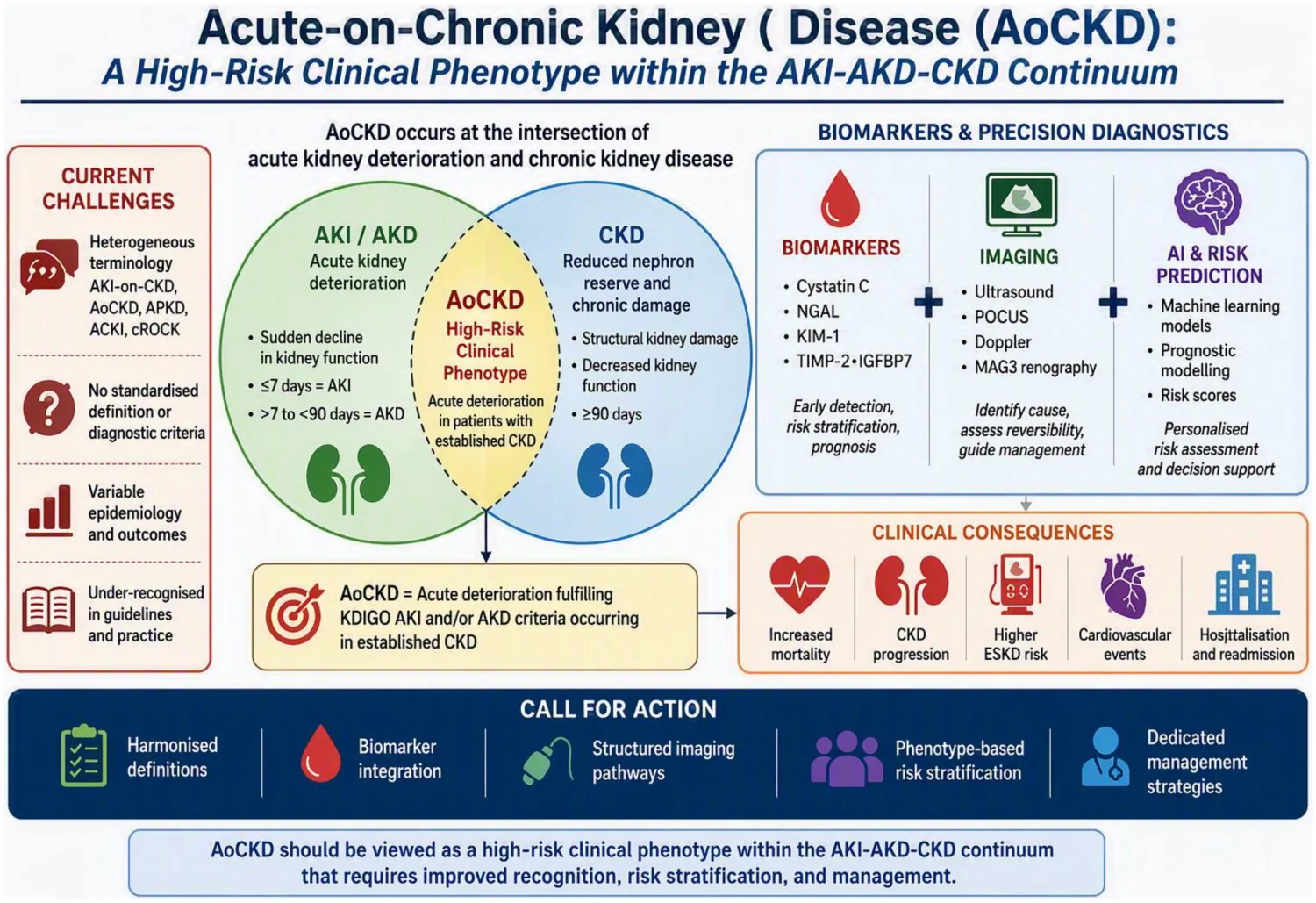
Visual abstract.

AoCKD is frequently misclassified as progressive CKD, even though timely recognition of acute deterioration in CKD offers a crucial opportunity for intervention, risk mitigation, and prevention of irreversible decline. Table 2 highlight the gaps in recognition of AoCKD in major guidelines.

**Table 2.** AoCKD recognition and gaps in major guidelines.

Organization	How they recognize AoCKD	What is recognized	Main gap
KDIGO	Does not define AoCKD as separate; acute CKD decline seen as AKD.	AKD <3 months including AKI and non-AKI >7 days.	No criteria for AoCKD.
NICE (NG203)	Does not recognize AoCKD or AKD; acute decline managed as AKI.	CKD defined by GFR/ACR; AKI follow-up.	No intermediate category; no AoCKD pathway.
ADQI	Does not use AoCKD term but overlaps.	AKD = persistent dysfunction 7–90 days after AKI.	Does not classify acute decline in CKD unless post-AKI.

This review examines current definitions, epidemiology, biomarker limitations, and research gaps related to AoCKD. We synthesize evidence from systematic reviews, registry data, consensus statements, and clinical studies, with particular attention to KDIGO–ADQI harmonization efforts, the prognostic value of eGFR variability, and the emerging role of cystatin C and other biomarkers [11]. We also highlight the lack of harmonization in the markers used to define AKI, AKD, and CKD—where creatinine‑based criteria dominate despite growing evidence supporting cystatin C–based eGFR.

One of the major unresolved challenges across the AKI-AKD-CKD spectrum is the integration of biomarkers in the definitions of kidney disease. Recent KDIGO and ADQI initiatives have made substantial progress toward harmonizing terminology and establishing a continuum of kidney disorders.

The KDIGO 2026 AKI/AKD guideline draft expands the role of cystatin C and kidney injury biomarkers in diagnosis, risk stratification, and kidney function assessment [8]. However, current AKI and AKD definitions remain primarily based on serum creatinine, urine output, and creatinine-derived estimates of kidney function [8,12]. Although cystatin C-based equations are increasingly recognized as more accurate measures of kidney function in selected populations, they have not yet been incorporated into operational AKD diagnostic criteria [9]. Consequently, differences persist between AKI/AKD and contemporary CKD frameworks, the latter increasingly favoring combined creatinine-cystatin C assessment [10].

## Methodology

This article is a state-of-the-art narrative review synthesizing current evidence, guideline frameworks, and conceptual models related to acute-on-chronic kidney disease (AoCKD). The aim was to evaluate definitional heterogeneity, epidemiologic variability, biomarker limitations, imaging gaps, and research priorities within the evolving AoCKD literature.

## Literature search and evidence identification

To inform this review, targeted literature searches were conducted in PubMed, Embase, and the Cochrane Library. Searches focused on identifying influential publications, clinical practice guidelines, consensus statements, systematic reviews, and observational studies relevant to acute deterioration in patients with established chronic kidney disease (CKD).

Search terms included combinations of:

- “Chronic kidney disease” OR “CKD”
- “Acute kidney injury” OR “AKI”
- “acute kidney disease” OR “AKD”
- “acute-on-chronic kidney disease”
- “acute-on-chronic renal failure”
- “rapid progression”
- “abrupt decline”
- “renal function deterioration”

Additional references were identified through citation tracking and review of key guideline documents, including publications from KDIGO, NICE, and the American College of Radiology–National Kidney Foundation (ACR–NKF).

## Evidence selection and synthesis

This review was conducted as a targeted narrative appraisal rather than a systematic review. Accordingly, evidence was selected on the basis of clinical relevance, influence on current practice, and contribution to the conceptual understanding of AoCKD. Formal systematic review procedures, including predefined inclusion criteria, duplicate screening, PRISMA flow diagrams, and reporting of records screened, were not undertaken.

Information extracted included terminology, diagnostic thresholds, epidemiological data, biomarker performance, imaging considerations, and proposed disease frameworks. Findings were synthesized narratively with emphasis on comparison of definitions, identification of inconsistencies, and areas requiring future harmonization and validation.

### Comparative epidemiology of AKI, AKD, and AoCKD

Acute kidney injury (AKI) is a common clinically significant syndrome with substantial global impact. Current estimates suggest that AKI affects more than 13 million people per year, contributing to approximately 1.7 million deaths worldwide [13]. Incidence varies considerably across clinical settings, with the highest rates observed in intensive care units, where AKI may occur in up to 67% of critically ill patients [14].

In the United Kingdom, the 2023 UK Renal Registry reported 681,127 AKI episodes among 586,734 patients, with nearly two‑thirds occurring in adults aged 65 years or older. Most AKI episodes are classified as stage 1 (71% at peak), and majority of patients experience only a single episode within a given year [15].

The epidemiology of AKD shows wide variability, depending on population characteristics and study design. Meta-analyses and large cohort studies report that around 1% to 33% develop AKD post AKI [16].

For example, Wang et al. identified 33% incidence of AKD in a cohort of 56,906 patients, while See et al. reported only 1% in a population with preserved baseline kidney function. Surgical populations demonstrated similarly broad variation, with AKD rates of 8% in orthopedic procedures and up to 30% in vascular or cardiac surgeries. Sepsis-associated AKD appear particularly common, with reported rates of 46.9% to 53.9%. The incidence of AKD occurring after AKI has been increasing over the last years [17]. This may be attributed to better recognition and coding. Age is a major determinant of risk with incidence approximately doubling with each additional decade of life [17].

In contrast, the epidemiology of acute‑on‑chronic kidney disease (AoCKD) remains poorly characterized largely because AoCKD lacks a formal definition in major guidelines. However, observational studies consistently show that patients with pre‑existing CKD who experience an acute deterioration—whether labeled as AKI, AKD, or ‘AKI on CKD’—represent the highest‑risk subgroup. These patients develop markedly increased risk of: CKD progression, end‑stage kidney disease (ESKD), cardiovascular events, hospitalization, readmission and Mortality [18–20]. The CKD-REIN study findings support the significant contribution of AoCKD to adverse cardiovascular outcomes [18,20].

The absence of a standardized definition of AoCKD has resulted in inconsistent classification across studies, limiting the ability to quantify its true incidence and burden. However, the available evidence strongly suggests that AoCKD is both common and clinically significant, underscoring the need for its explicit inclusion recognition in future guideline frameworks.

The true incidence of AoCKD remains difficult to determine and may be underestimated in existing studies. Much of the available literature has classified patients using broader AKI or AKD frameworks without specifically identifying individuals with preexisting CKD who experience acute deterioration. Furthermore, substantial heterogeneity exists in the diagnostic criteria used across studies, including differences in serum creatinine thresholds, definitions of baseline kidney function, and duration of follow-up. The evolving role of cystatin C and cystatin C-based estimated glomerular filtration rate (eGFR) further complicates epidemiological comparisons. While recent KDIGO guidance has expanded the role of cystatin C in kidney disease assessment and risk stratification, formal AKI and AKD definitions remain largely centered on creatinine-based measures.

Consequently, differing biomarker strategies may identify different patient populations, contributing to variability in reported incidence estimates. Until harmonized definitions and biomarker frameworks are established, reliable estimates of the true burden of AoCKD will remain challenging.

Accordingly, the available evidence suggests that AoCKD is a common and clinically significant condition; however, its true incidence remains uncertain because of ongoing heterogeneity in definitions, biomarkers, and study methodologies.

## Definitions ambiguity

A variety of terms and acronyms are used in the literature to describe acute deterioration in patients with established chronic kidney disease (CKD), Table 3. These include *acute‑on‑chronic kidney disease (AoCKD)*, *AKI‑on‑CKD*, *acute on pre‑existing kidney disease (APCD)*, *acute on chronic kidney injury (ACKI)*, and *acute decompensation of CKD (AD‑CKD)*. Such labels appear inconsistently across clinical reviews, observational studies, and educational resources, yet none are formally defined in KDIGO or ADQI guidelines. The coexistence of multiple non‑standardized terms reflects the underlying conceptual ambiguity surrounding this clinical state and highlights the need for a unified, guideline‑level definition.

**Table 3.** Various definitions suggested for AoCKD in literature.

Acronym	Source	Definition criteria	Outcome/predicted mortality	Weakness
Acute preexisting kidney disease (APKD) [21,22]	Sun et al. 2023; Hong et al. 2023	‘Acute on preexisting kidney disease’: AKI defined by APKD criteria in CKD population	Better mortality correlation than KDIGO in CKD patients (Hong et al. 2023) Offers better alternative to KDIGO criteria when there is no baseline value between 7–365 days	Criteria were developed for critical care patients The abbreviation of APKD overlaps and acquired polycystic kidney disease
Acute chronic kidney injury (ACKI) cROCK criteria	Data from >711000 patients record [22,23]	The criteria are based on reference change value in serum creatinine (RCV) cROCK. ACKI is defined as *a* ≥ 25% increase in SCr in 7 days	cROCK detected 40% more ACKI events than KDIGO cROCK criteria have detected mortality better than KDIGO	Requires more wide validation through different races
AKI-on-CKD	KDIGO (2012 AKI definition applied to CKD)	KDIGO AKI stages (SCr/UO changes) applied in patients with established CKD	Straightforward use of existing KDIGO metrics	KDIGO criteria showed poor mortality correlation in CKD cohort Does not adjust for baseline CKD

cROCK serum creatinine optimized criteria for acute kidney injury in CKD.

Despite the lack of consensus terminology, several core themes recur across the literature. AoCKD represents a high-risk clinical phenotype in which an acute kidney insult occurs on a background of reduced nephron mass, impaired autoregulation, and increased susceptibility to haemodynamic, inflammatory, or nephrotoxic stressors. Importantly, the prognostic implications of AoCKD differ meaningfully from those of acute kidney injury (AKI) in individuals with previously normal renal function.

Mortality patterns further illustrate this divergence. In patients with CKD, KDIGO stage 2 AKI may confer a higher mortality risk than stage 3 AKI, suggesting that current staging systems—developed primarily for AKI in structurally normal kidneys—may inadequately capture risk within the AoCKD population [21]. This observation reinforces the need for CKD‑specific risk stratification or modified diagnostic criteria.

Several groups have attempted to develop definitions tailored to acute deterioration in CKD. The cROCK criteria, for instance, classify acute worsening as *a* ≥ 25% rise in serum creatinine within 7 days. Derived from more than 711,000 patient records, these criteria have demonstrated greater sensitivity for detecting clinically meaningful AKI in CKD cohorts compared with the KDIGO 2012 definition [22]. The report acknowledges that even modest absolute rises in creatinine may represent substantial functional decline when baseline GFR is reduced, and they correlate more closely with patient outcomes than KDIGO thresholds.

Despite these advances, no major guideline—including KDIGO, ADQI, or NICE—has formally endorsed a definition for AoCKD. Consequently, AoCKD remains inconsistently recognized, and frequently misclassified as progressive CKD rather than an acute episode superimposed on chronic disease. This definitional gap hinders early detection, risk assessment, epidemiological accuracy, and the development of targeted therapeutic interventions.

## Proposed operational definition of acute-on-chronic kidney disease (AoCKD)

Acute-on-chronic kidney disease (AoCKD) describes an acute or subacute deterioration in kidney function and/or structure occurring in an individual with established chronic kidney disease (CKD). Established CKD is defined according to KDIGO criteria as abnormalities of kidney structure or function present for at least 3 months.

AoCKD may be recognized when established CKD is accompanied by one or more of the following:

- Functional deterioration
- A ≥25% increase in serum creatinine from an established baseline within 7 days, consistent with the cROCK concept; or
- An acute decline in estimated glomerular filtration rate (eGFR) exceeding expected biological and analytical variability; or
- Fulfillment of conventional KDIGO functional criteria for acute kidney injury (AKI).
- Structural kidney injury
- New evidence of kidney injury occurring over an acute or subacute timeframe, including new or worsening albuminuria, hematuria, or validated kidney injury biomarkers, despite insufficient functional change to fulfill conventional AKI criteria.
- Duration
- The deterioration should occur over an acute or subacute period (≤90 days), thereby distinguishing AoCKD from the expected course of chronic CKD progression.

AoCKD should be regarded as a proposed phenotype-based framework encompassing subthreshold functional deterioration, conventional AKI superimposed on CKD, and structural kidney injury in patients with established CKD. The construct is intended to facilitate clinical phenotyping and research and requires prospective validation before routine clinical implementation.

## Hemodynamic eGFR declines following RAAS and SGLT2 inhibitor initiation

An important diagnostic consideration is the distinction between true AoCKD and the expected haemodynamic decline in kidney function that may occur following initiation of renin–angiotensin–aldosterone system (RAAS) inhibitors or sodium-glucose cotransporter-2 (SGLT2) inhibitors. These therapies reduce intraglomerular pressure and may cause an early decline in estimated glomerular filtration rate (eGFR), which is generally transient, stabilizes over time, and is associated with long-term renoprotective benefits.

Accordingly, an isolated reduction in eGFR occurring shortly after initiation or dose escalation of RAAS inhibitors or SGLT2 inhibitors should not automatically be classified as AoCKD. Interpretation should incorporate the temporal relationship to treatment initiation, volume status, blood pressure, albuminuria, and evidence of structural kidney injury. Progressive creatinine elevation, persistent kidney dysfunction, haemodynamic instability, or concomitant markers of kidney injury may suggest superimposed AoCKD rather than an expected haemodynamic response.

When evaluating suspected AoCKD, recent initiation or intensification of RAAS inhibitor or SGLT2 inhibitor therapy should be considered an important differential diagnosis and routinely assessed as part of the medication review.

C Established CKD

↓

Acute departure from CKD trajectory

↓

AoCKDwith overlapping phenotypes:

- AoCKD below AKI threshold
- AoCKD meeting AKI criteria
- AoCKD with structural injury/biomarker positivity
- AoCKD progressing to AKD

## Outcomes: mortality, CKD progression, and cardiovascular events

A substantial body of evidence demonstrates that any acute deterioration in kidney function—whether meeting criteria for acute kidney injury (AKI), acute kidney disease (AKD), or representing an acute event superimposed on chronic kidney disease (AoCKD)—carries significant long‑term consequences. Meta‑analyses consistently show that AKI markedly increases the risk of adverse renal and cardiovascular outcomes.

Patients who experience AKI are at substantially increased risk of adverse long-term kidney outcomes. A recent systematic review and meta-analysis found that AKI was associated with a higher risk of CKD progression (HR 1.83, 95% CI 1.26–2.40), kidney failure (HR 2.64, 95% CI 2.03–3.25), and major adverse kidney events (MAKE) (OR 2.77, 95% CI 2.01–3.53) compared with individuals without AKI [24,25].

The impact of AKD on mortality is similarly substantial. with reported hazard 3.8 (95% CI, 2.2–6.7), Acute kidney disease (AKD) is also associated with a substantially increased risk of CKD progression. In a systematic review and meta-analysis involving more than one million patients, AKD was associated with a more than fourfold higher likelihood of incident or progressive CKD compared with patients without AKD (OR 4.22, 95% CI 3.38–5.27). AKD was also associated with higher risks of end-stage kidney disease (OR 6.58, 95% CI 4.67–9.27) and all-cause mortality (OR 3.62, 95% CI 2.64–4.95) [6,26].

Although AoCKD is not formally defined in current guidelines, emerging evidence strongly suggests that it represents the highest‑risk phenotype across the spectrum of kidney disorders. Patients with underlying CKD who experience an acute deterioration display disproportionately high rates of irreversible CKD progression, progression to end‑stage kidney disease, recurrent hospitalization, major adverse cardiovascular events, and all‑cause mortality [18,19]. This amplified risk likely reflects the combined burden of chronic structural damage, reduced nephron reserve, impaired autoregulatory capacity, and heightened vulnerability to haemodynamic or nephrotoxic insults.

### AoCKD recognition in KDIGO and NICE guidelines

#### KDIGO guidelines

KDIGO has established internationally accepted definitions for AKI, AKD, and CKD but does not currently recognize acute-on-chronic kidney disease (AoCKD) as a distinct diagnostic entity. The AKD concept was introduced in the 2012 KDIGO AKI guideline and subsequently formalized by the ADQI 16 Workgroup. More recently, the KDIGO Consensus Conference on Harmonizing Acute and Chronic Kidney Disease Definitions and Classification proposed an integrated framework linking AKI, AKD, and CKD across the spectrum of kidney disease. Under this framework, AKD may occur with or without AKI and may coexist with preexisting CKD.

The draft KDIGO 2026 AKI/AKD Guideline further defines AKD as abnormalities of kidney function and/or structure of ≤3 months’ duration, including AKI, reduced glomerular filtration rate, substantial declines in kidney function, increases in serum creatinine, or markers of kidney damage. Despite these advances, acute deterioration occurring in patients with established CKD is not separately classified and remains subsumed within the broader AKD framework, (Table 2).

Accordingly, the proposed AoCKD construct should be viewed as a complementary phenotype-based framework describing an acute or subacute departure from an established CKD trajectory rather than as an alternative to existing KDIGO AKI or AKD definitions.

### NICE guidelines

The NICE Guideline NG203 for chronic kidney disease provides recommendations for CKD diagnosis, risk stratification, monitoring, and referral based primarily on estimated glomerular filtration rate (eGFR) and albuminuria categories [27]. Complementing this, NICE Guideline NG148 addresses the prevention, detection, and management of acute kidney injury (AKI), including post-AKI follow-up and medication review [28].

However, neither guideline recognizes acute-on-chronic kidney disease (AoCKD) as a distinct diagnostic entity or provides specific diagnostic criteria for acute deterioration occurring in patients with established CKD. Instead, such presentations are managed within existing AKI and CKD pathways [27].

## ACR–NKF consensus statement on intravenous iodinated contrast media

The American College of Radiology–National Kidney Foundation (ACR–NKF) Consensus Statement on intravenous iodinated contrast media highlighted that the risk of contrast-associated acute kidney injury has historically been overestimated, particularly with modern low-osmolar contrast agents. The statement emphasized individualized risk assessment and recommended prophylactic intravenous hydration primarily for patients with AKI or severe CKD (eGFR <30 mL/min/1.73 m^2^). Although the document does not specifically address acute-on-chronic kidney disease (AoCKD), it provides important guidance regarding a common precipitating factor for acute deterioration in kidney function among patients with CKD [29].

## eGFR variability in CKD patients as a prognostic marker

### Prognostic value of eGFR variability and decline

Changes in estimated glomerular filtration rate (eGFR), including acute declines and increased variability, are associated with adverse kidney outcomes in patients with chronic kidney disease (CKD). Substantial reductions in eGFR following acute kidney disease (AKD) have been linked to an increased risk of CKD progression, kidney failure, and mortality [6,30].

Similarly, discordance between cystatin C-based estimated glomerular filtration rate (eGFRcys) and creatinine-based estimated glomerular filtration rate (eGFRcr) has emerged as an important prognostic marker. A recent systematic review and meta-analysis demonstrated that individuals with lower eGFRcys relative to eGFRcr had a higher risk of all-cause mortality (HR 1.58, 95% CI 1.42–1.76) and cardiovascular events (HR 1.32, 95% CI 1.25–1.39) [31,32].

## Clinical implications

These findings support the role of eGFR variability as prognostic markers and potential surrogate endpoints in clinical research. However, the distinction between expected CKD progression and clinically significant acute deterioration remains poorly defined. The absence of standardized thresholds for eGFR variability and eGFR discordance limits their incorporation into routine practice and clinical guidelines, highlighting the need for further validation and consensus.

## Estimation of baseline kidney function in AKI superimposed on CKD

Accurate determination of baseline kidney function remains a major challenge when diagnosing acute kidney injury (AKI) in patients with preexisting chronic kidney disease (CKD). Reliance on a single historical serum creatinine value may result in misclassification, particularly in individuals with fluctuating kidney function or limited prior laboratory data. Recent approaches have sought to improve baseline kidney function estimation and AKI classification using longitudinal kidney function trajectories and reference change values. In this context, Larsen et al. reported a modest net reclassification improvement of 2.5% using an alternative approach to baseline kidney function assessment, highlighting the potential value of more individualized methods for AKI detection in CKD populations [33].

However, differentiating true AKI from expected CKD progression or other forms of acute-on-chronic kidney deterioration remains challenging when reliable baseline kidney function data are unavailable. Improved methods for baseline kidney function estimation may facilitate more accurate identification of acute kidney events in patients with established CKD and support future validation of acute-on-chronic kidney disease (AoCKD) as a distinct clinical phenotype.

## Role of cystatin C and cystatin C–based eGFR in predicting morbidity and mortality

### Biomarker performance for detection of AKI, AKD and AoCKD

A panel of urinary biomarkers have been approved by the Food and Drug Administration (FDA) for early detection of drug induced AKI [34]. Table 4 compares the performance of various biomarkers for AKI and CKD [23,36–38]. It is worth noting that different biomarkers differ in sensitivity, specificity and limitations [39–49].

**Table 4.** Selected biomarkers and functional markers relevant to AKI, AKD, CKD and AoCKD.

Bomarker/marker	Primary role	Strengths	Limitations	Potential relevance to AoCKD	Key reference
Serum Creatinine [8]	Functional marker	Widely available; basis of KDIGO AKI criteria	Delayed rise; affected by muscle mass and non-renal factors	Standard marker for detecting functional deterioration in AoCKD	KDIGO AKI Guideline
Urine Output [8]	Functional marker	Early indicator of AKI	Poor specificity; often unavailable outside hospital	Supports recognition of AKI superimposed on CKD	KDIGO AKI Guideline
Cystatin C [35]	Functional marker of GFR	Less affected by muscle mass; detects changes earlier than creatinine in some settings	Affected by inflammation, thyroid disease, corticosteroids; limited availability	May detect acute deterioration before conventional creatinine-based AKI	KDIGO 2024 CKD Guideline
Albuminuria (uACR) [35]	Structural marker	Established prognostic marker in CKD	Non-specific; affected by exercise, infection, diabetes	May indicate structural kidney injury even without AKI criteria	KDIGO 2024 CKD Guideline
Hematuria [8]	Structural marker	Easily measured	Limited specificity	May support evidence of acute structural injury in AoCKD	KDIGO 2026 AKI/AKD Draft
NGAL [36]	Tubular injury biomarker	Early marker of tubular injury	Influenced by inflammation and sepsis	May identify structural injury before creatinine rise	Haase et al.
KIM-1 [37]	Tubular injury biomarker	Reflects proximal tubular injury	Limited routine availability	Potential marker of subclinical AoCKD	Vaidya et al.
TIMP-2•IGFBP7 [38]	Cell-cycle arrest biomarker	FDA-cleared for AKI risk assessment	Primarily prognostic rather than diagnostic	May identify patients at risk of AoCKD progression	Kashani et al.
eGFRcr-cys Discordance [35]	Risk stratification marker	Associated with adverse kidney and cardiovascular outcomes	No universally accepted threshold	May identify higher-risk CKD patients susceptible to AoCKD	KDIGO 2024 CKD Guideline

Abbreviations: AKI, acute kidney injury; AKD, acute kidney disease; AoCKD, acute-on-chronic kidney disease; CKD, chronic kidney disease; eGFR, estimated glomerular filtration rate; NGAL, neutrophil gelatinase-associated lipocalin; KIM-1, kidney injury molecule-1; TIMP-2, tissue inhibitor of metalloproteinase-2; IGFBP7, insulin-like growth factor-binding protein 7; uACR, urine albumin-to-creatinine ratio.

Cystatin C has emerged as a superior biomarker for estimating GFR, particularly in populations where there could be fallacies in creatinine based eGFR with low muscle mass, elderly patients, and those with critical illness. Studies consistently show that cystatin C-based eGFR

Meta-analyses and large cohort studies (e.g., UK Biobank, SCREAM, ARIC) demonstrate that individuals with lower eGFRcys relative to eGFRcr have significantly higher risks of mortality and cardiovascular events, with hazard ratios ranging from 1.25 to 1.58 for mortality and 1.32 for cardiovascular events.

In one prospective study, it has been found that cystatin C better correlated with CKD after AKI stage 3 in patients in the intensive care unit (ITU). It has also been correlated with long term mortality [42,44].

Furthermore, the growing recognition of structural kidney injury biomarkers highlights potential opportunities for earlier diagnosis before substantial changes in serum creatinine occur. Nevertheless, interpretation of these biomarkers in CKD remains challenging due to baseline elevations, heterogeneity of underlying kidney disease, and limited validation across diverse CKD populations. Additional research is therefore required to establish optimal thresholds and phenotype-specific approaches for patients experiencing acute deterioration on a background of CKD.

## Influence of CKD aetiology on biomarker performance

Interpretation of biomarkers in patients with acute deterioration superimposed on CKD may be influenced by the underlying cause of kidney disease. In diabetic kidney disease, chronic tubular injury, inflammation, and albuminuria may lead to persistently elevated biomarker levels, potentially reducing specificity for the detection of superimposed acute injury. Patients with hypertensive nephrosclerosis may exhibit predominantly vascular and interstitial changes, whereas those with glomerular diseases may demonstrate biomarker elevations related to ongoing immune-mediated inflammation independent of acute functional decline. Consequently, biomarker thresholds and diagnostic performance may vary according to CKD phenotype, highlighting the need for disease-specific validation studies and precision medicine approaches when applying biomarkers to patients with AKI, AKD, and AoCKD.

## Cost-effectiveness and implementation challenges

Despite the promising diagnostic performance of biomarkers such as NGAL, KIM-1, and TIMP-2•IGFBP7, widespread implementation remains constrained by cost, laboratory availability, and uncertainty regarding cost-effectiveness in routine clinical practice. Future studies should evaluate not only diagnostic accuracy but also clinical effectiveness, health-economic impact, and integration within existing care pathways before broader adoption can be recommended.

## Call for action to embed biomarkers panel in the diagnosis and prognosis of AoCKD

There is growing evidence for cystatin C as prognostic marker for AKI However its integration into AKD and AoCKD definitions and staging proposals remains limited.

KDIGO 2024 guidelines recommend using eGFRcr-cys in clinical situations where eGFRcr is less accurate and GFR affects clinical decision-making, but do not mandate cystatin C for AKD or AoCKD diagnosis [35].

Recent studies suggest that eGFR discordance (eGFRcys-eGFRcr < −15 mL/min/1.73 m^2^) could serve as a marker for identifying vulnerable populations at higher risk of adverse outcomes, and that incorporating cystatin C into risk prediction models enhances accuracy.

We call for action to review the evidence and embed the biomarker panels as early markers of AoCKD, especially where there are fallacies in the routinely used markers. we also call for actions to embed them as prognostic markers and uses them to risk stratify patients with AoCKD and tailor the necessary interventions.

## Gaps in imaging and functional tests for AKD and AoCKD detection and prognosis

The recently published KDIGO 2026 Clinical Practice Guideline for Acute Kidney Injury (AKI) and Acute Kidney Disease (AKD) represents an important advance in harmonizing definitions across the spectrum of kidney disorders and broadens the role of biomarkers in diagnosis and classification[8]. Nevertheless, imaging remains relatively underrepresented compared with biochemical markers and functional measures. Whilst the updated guideline acknowledges the importance of kidney structure assessment and recognizes the complementary role of imaging in diagnostic evaluation, imaging findings are not incorporated into formal AKI or AKD staging systems, risk stratification frameworks, or management algorithms.

The KDIGO 2026 guideline continues to rely primarily on changes in serum creatinine, cystatin C where appropriate, urine output, and markers of kidney injury for the diagnosis and classification of AKI and AKD. Although an integrated approach to the assessment of kidney function and structure is encouraged, guidance regarding the optimal use of ultrasound, Doppler studies, point-of-care ultrasound (POCUS), or functional imaging modalities remains limited. In particular, there is little specific guidance addressing patients with established CKD who develop acute deterioration in renal function, a population in whom differentiation between reversible injury and chronic progression may be particularly challenging.

The limited integration of imaging into current AKI and AKD frameworks highlights an important area for future research. Prospective studies are needed to determine whether incorporating structural and functional imaging parameters into diagnostic and prognostic pathways can improve risk stratification and management of patients experiencing acute deterioration on a background of CKD.

Similarly, the KDIGO AKI/AKD guideline update scope and the 2020 consensus work on harmonizing AKI, AKD, and CKD definitions focus on classification and care pathways but do not develop imaging‑based algorithms or modality‑specific recommendations for AKD or AoCKD, despite the central role of ultrasound, CT/MRI, and nuclear medicine in distinguishing reversible acute pathology from irreversible chronic damage [50].

This leaves important gaps: there is no guidance on when to seek cross‑sectional or functional imaging, no discussion of scintigraphy (e.g., MAG3) for differentiating obstructive from non‑obstructive hydronephrosis or assessing differential function in AoCKD [51,52], Table 5 and no structured advice on balancing diagnostic yield against contrast‑associated risk in patients with reduced GFR, even though contrast exposure during AKI has been associated with worse renal outcomes at higher cumulative doses.

**Table 5.** Gaps in major guidelines recommendations for imaging in AKD/AoCKD.

Guideline	Role of imaging	Potential caveats
*KDIGO-AKI definition	Ultrasound mentioned in the stage-based approach only	No mention of the functional imaging No dose specifications to prevent contrast induced nephropathy in patients with CKD (only mentions use the lowest dose
ADQI 16 Workgroup**	AKD definition and staging are based primarily on the creatine values	As above No clear role of functional or structural images in determinations of AKD
Harmonizing AKD and CKD	For AKD: Mentions Duplex Doppler ultrasound Mentions kidneys size on ultrasound as supportive for diagnosis of AKD Ultrasound if suspicion of obstruction Also mentions CT, MRI States recommendation to consider alternative to radiocontrast (moderate)	No dose specification for contrast use or recommendation to use calculators or cutoff value for kidney function No mention of contrast-enhanced ultrasound (CEUS) as alternative to iodinated contrast No specification of when to use the functional or advanced imaging modalities in AKD Advanced modalities (FDG-PET, diffusion-weighted MRI, elastography) not addressed

** Acute kidney disease and renal recovery: consensus report of the Acute Disease Quality Initiative (ADQI) 16 Workgroup.

PET scan: positron emission tomography.

Multiorgan point of care ultrasound (POCUS) provides an opportunity to assess the body hemodynamic and to assess the inflow and outflow of the kidneys which can help identify vascular underfilling and importantly the venous congestion. this can help aid decision making on volume resuscitation in patients with AKI, AKD and AoCKD [53]. It also help ameliorate the deleterious effect of volume overload on AKI and AKD progression. however, its role in the guidelines is not substantiated [54,55]. POCUS can be invariable in managing acute on chronic cardio-renal syndrome [56]

The choice of imaging modality in the context of AKD and AoCKD remains largely practice‑pattern driven rather than guideline‑directed. This gap calls for more work to render the major guidelines inclusive on recommendations to integrate structural and functional imaging into diagnostic and management pathways.

## Call for action: how guidelines could improve recommendations on imaging in the context of AKD/AoCKD

The review supports efforts to improve the utilization of images in the context of AKD and AoCKD. Ideally imaging should serve to differentiate reversible declines of kidney function from progressive decline in CKD. this will allow timely interventions to mitigate the risks. Table 6 summarizes the suggested recommendations for image inclusive guidelines.

**Table 6.** Proposed enhancements to incorporate roles of imaging in AKI, AKD and AoCKD.

Formulation of diagnostic algorithm for AKD, AKI and AoCKD Clarification of the dose of contrast than can be used in at different stages of AKD and AoCKD Clarification and inclusion of the role of functional images MAG3/DTPA in obstruction assessment, to assess kidney reserve in AoCKD.
synthesize evidence to support use of ‘Novel ultrasound techniques (elastography, CEUS)’ Clarification of the role of POCUS in AKD and AoCkd
MAG3 mercaptoacetyltriglycine) renogram DTPA scan nuclear medicine imaging test using a radioactive tracer (Technetium-99m DTPA)

While conventional ultrasonography remains the basic modality to assess the cortical thickness, kidney size and bladder outflow, advanced in functional and cross-sectional images have allowed more precision for assessment of AKI, CKD. Evidence and cost benefit should be synthesized.

There is a need to better define what should be the next step imaging techniques. examples are non‑contrast CT for suspected stones, retroperitoneal fibrosis, or masses., Doppler US ± CTA/MRA for suspected renovascular disease, with explicit consideration of CKD stage and contrast risk. Functional imaging**:** MAG3 renography for unresolved hydronephrosis, suspected obstruction on CKD background, and differential function to guide intervention decisions.

POCUS and multiorgan POCUM should be properly based in the guidelines as invaluable bedside tool to optimize management of AKI, AKD and AoCKD, especially used in acute on chronic cardiorenal syndrome

Recommendations on the safety of use of contrast images in the context of AKI, AoCKD may benefit from more specification. Most guidelines generally recommended to avoid contrast in the context of AKI; the recommendations are not clear as whether contrast images could be used in AKD or AoCKD. Recommendations may include the allowed maximal dose of iodinated contrast that could be used and whether any early urinary or serum biomarkers can be used to predict deterioration of kidney functions [57,58].

## Practical management perspectives for AoCKD

Table 6 highlights the practical management strategies for patients with acute on chronic kidney disease (AoCKD).

Figure 2 shows Proposed Operational Framework for Acute-on-chronic kidney disease (AoCKD) diagnosis and management.

**Figure 2.**
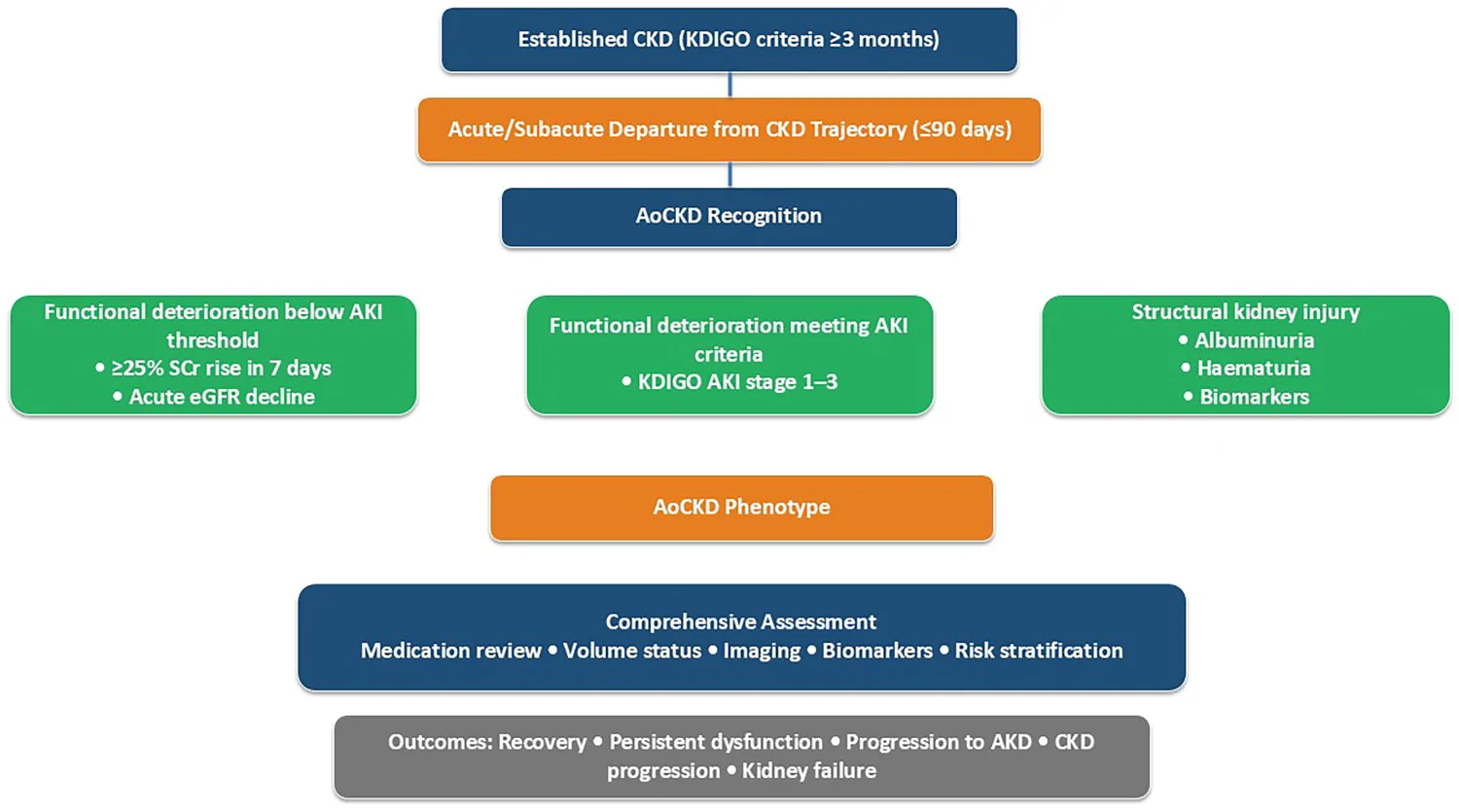
Proposed operational framework for acute-on-chronic kidney disease (AoCKD) diagnosis and management (expert opinion). AoCKD is proposed as an acute or subacute departure from an established chronic kidney disease (CKD) trajectory occurring over ≤90 days. Recognition may be based on: (i) functional deterioration below conventional acute kidney injury (AKI) thresholds, including a ≥25% increase in serum creatinine from baseline within 7 days (cROCK concept) or an acute decline in estimated glomerular filtration rate (eGFR) exceeding expected biological and analytical variability; (ii) deterioration fulfilling KDIGO AKI criteria; or (iii) evidence of structural kidney injury, including new or worsening albuminuria, hematuria, or validated kidney injury biomarkers. Following recognition, comprehensive clinical assessment is undertaken to identify reversible causes, stratify risk, and guide management. Potential clinical trajectories include recovery, persistent dysfunction, progression to acute kidney disease (AKD), CKD progression, or kidney failure. This figure represents the authors’ expert opinion and a proposed phenotype-based operational framework intended to facilitate clinical phenotyping and future research in AoCKD. The framework is not currently recognized by KDIGO, NICE, or other clinical practice guidelines and requires prospective validation before routine clinical implementation. Abbreviations: AoCKD, acute-on-chronic kidney disease; AKI, acute kidney injury; AKD, acute kidney disease; CKD, chronic kidney disease; cROCK, clinical reference change value for kidney function; eGFR, estimated glomerular filtration rate; KDIGO, Kidney Disease: Improving Global Outcomes.

Table 7 also lists the practical management complications (Expert opinion).

**Table 7.** Practical management considerations in AoCKD (expert opinion).

Domain	Suggested action
Medication review	Stop nephrotoxins
Volume status	Assess using clinical examination and POCUS
Biomarkers	Creatinine plus cystatin C where appropriate
Imaging	Ultrasound ± functional imaging
Blood pressure	Individualized optimization
Sick-day guidance	Patient education
Follow-up	Repeat kidney function monitoring
Referral	Early nephrology input for high-risk cases

## Precision medicine and machine 7 gaps in AKI, AKD, and AoCKD guidelines

Current guidance for AKI and AKD remains centered on late conventional biochemical markers—principally serum creatinine and urine output—despite major advances in molecular diagnostics and computational imaging. This can be limiting in acute‑on‑chronic kidney disease (AoCKD), where clinicians must rapidly distinguish reversible acute injury from chronic structural decline. Precision‑medicine may offer a more dynamic approach by integrating tubular injury biomarkers (NGAL, KIM‑1, L‑FABP, IL‑18), cell‑cycle arrest markers (TIMP‑2·IGFBP7), and filtration markers (cystatin C), all of which can detect renal stress hours before creatinine rises [59]. This will allow tailoring investigation tools and management to patients’ phenotypes rather than one size fit all model of current guidelines.

These biomarkers have proven predictive value for AKI severity, need for renal replacement therapy, and short‑term outcomes across perioperative, critical‑care, and contrast‑induced [58,60] nephropathy.

Integrating early biomarkers detection with structured imaging diagnostic pathways can improve diagnostic yield and mitigate the adverse outcomes the concept of precision medicine is poorly represented in current guidelines. Integrating biomarker‑guided imaging into future guideline updates would reshape concepts around AKI/AKD/AoCKD management and align recommendations with contemporary diagnostic capabilities.

## Deep machine learning

DL models have demonstrated meaningful performance in automating renal ultrasound interpretation, detecting hydronephrosis, cortical thinning, and Doppler abnormalities, and analyzing MAG3 time‑activity curves with improved reproducibility [61,62]. Multimodal ML models that combine imaging features, biomarkers, and electronic health record data can generate personalized predictions for renal recovery, dialysis requirement, and mortality—precisely the prognostic insights that current guidelines lack [63,64].

Yet, KDIGO provides no direction on how these tools should be validated, integrated, or governed. This omission risks widening the gap between real‑world practice and guideline recommendations as AI‑enabled imaging becomes increasingly common. Incorporating ML‑assisted triage, automated functional interpretation, and multimodal prognostic modeling into future guideline frameworks would address long‑standing deficits in early detection, risk stratification, and imaging standardization in AoCKD.

## Limitations

This review has several limitations. First, it was conducted as a targeted narrative review rather than a systematic review and therefore does not follow formal PRISMA methodology. Evidence selection was based on relevance, influence, and contribution to the conceptual understanding of acute-on-chronic kidney disease (AoCKD), which may introduce selection bias. Second, the proposed operational definition of AoCKD represents a hypothesis-generating framework derived from the available literature and expert interpretation rather than a validated diagnostic classification. Third, substantial heterogeneity persists across studies regarding the definitions of acute kidney injury (AKI), acute kidney disease (AKD), kidney recovery, and major adverse kidney events (MAKE), limiting direct comparisons between studies. Finally, several biomarkers and diagnostic approaches discussed remain investigational and require prospective validation before routine clinical implementation.

## Conclusion

Acute-on-chronic kidney disease (AoCKD) represents a high-risk clinical phenotype of renal injury, with high incidence, adverse outcomes, and substantial healthcare burden. Despite advances in the understanding of AKI, AKD, and CKD, AoCKD remains under-recognized in major guidelines, and its management is hampered by definitional gaps, biomarker limitations, and lack of integration into care pathways.

Recent evidence highlights the prognostic value of eGFR variability, cystatin C, and novel biomarkers, but standardization and validation are needed for routine clinical use. Imaging and functional tests offer promise for early detection and risk stratification but require further research. The underrepresentation of AoCKD in clinical trials and heterogeneity in outcome measures impede progress toward evidence-based interventions.

Addressing these gaps requires coordinated efforts in guideline development, research prioritization, and implementation of structured surveillance and multidisciplinary care. The integration of biomarker panels, standardized definitions, and inclusive trial designs will be essential for improving outcomes in this high-risk population.

## Key takeaways

AoCKD is a high-risk, under-recognized phase in the kidney disease continuum, with substantial adverse outcomes and healthcare implications. Current guidelines lack explicit definitions and management pathways, and biomarker limitations hinder early detection and risk stratification. Harmonization of definitions, validation of novel biomarkers, and integration of structured surveillance into CKD care are urgent priorities for research and clinical practice.

## Data Availability

All data generated or analyzed during this study are included in this published article.

## Compliance with the Helsinki Declaration

The principles discussed in this manuscript are in accordance with the ethical standards outlined in the Declaration of Helsinki.
